# A Novel Normalized Quantitative Real-Time PCR Approach for Ensuring Roe Deer (*Capreolus capreolus*) Meat Authenticity in Game Meat Foods

**DOI:** 10.3390/foods13233728

**Published:** 2024-11-21

**Authors:** Bukola M. Adenuga, Rita Biltes, Caterina Villa, Joana Costa, Anita Spychaj, Magdalena Montowska, Isabel Mafra

**Affiliations:** 1 LAQV/REQUIMTE, Faculdade de Farmácia, Universidade do Porto, Rua de Jorge Viterbo Ferreira, 228, 4050-313 Porto, Portugal; bukola.adenuga@up.poznan.pl (B.M.A.); rbmachado@ff.up.pt (R.B.); cvilla@ff.up.pt (C.V.); jbcosta@ff.up.pt (J.C.); 2 Department of Meat Technology, Poznań University of Life Sciences, ul. Wojska Polskiego 31, 60-624 Poznań, Poland; anita.spychaj@up.poznan.pl (A.S.); magdalena.montowska@up.poznan.pl (M.M.)

**Keywords:** food authentication, game meat products, roe deer, nuclear marker, agouti signaling protein (ASIP) gene, qPCR

## Abstract

Roe deer meat is a prized game product in many European countries. However, concerns exist regarding the accuracy of the amount of declared roe deer in processed game meat foods. This study aimed to develop a reliable method for the detection and quantification of roe deer in commercialized game meat products. A TaqMan probe-based quantitative real-time PCR (qPCR) assay was designed, targeting a single-copy 120-bp region of the roe deer agouti signaling protein (ASIP) encoding gene. The method employed the normalized ∆Cq approach to establish a calibration curve for roe deer detection and quantification within 0.05–50% (*w*/*w*) in complex raw and processed matrices. The method proved to be specific for roe deer identification, achieving limits of detection and quantification of 0.04 ng of roe deer DNA and 0.05% (*w*/*w*) of roe deer in simulated pâté. Following validation with blind samples, highlighting the precision and trueness of the approach, the assay was applied to 46 market samples from four European origins (Poland, Portugal, France, and Spain). The analysis revealed significant discrepancies between declared roe deer content and actual levels in all roe deer labeled products. The global analysis of results, combining the previous survey on red deer species with present roe deer data, identified 61% of mislabeled/adulterated samples due to the absence of deer species, substitution of roe deer with red deer, substitution of fallow deer with other deer species and red deer with pork, and undeclared addition of roe deer. This study demonstrates the effectiveness of the developed qPCR method for accurate roe deer meat authentication in foods, showing its usefulness as a tool for routine food inspection to ensure labeling compliance.

## 1. Introduction

Roe deer (*Capreolus capreolus*) is a highly valued game species in Europe, with an estimated population of 15 million individuals [1]. Its meat is prized for excellent flavor, texture, and nutritional value (134 calories, 21 g of protein, and 1.2 g of fat per 100 g serving portion) [2], contributing significantly to the economic value of the hunting industry in nations like Germany, the Czech Republic, Poland, France, Austria, Sweden, and Belgium [3]. In fact, roe deer hunting, combined with red deer and wild boar, results in a total economic value of more than 10 billion EUR, with substantial contributions from tourism, land management, and the sale of game meat [4]. Poland, in particular, is famous for its roe deer hunting season, in which hunters from European countries participate in ensuring the availability of roe deer venison in the Polish meat market. Despite extensive research on other aspects of roe deer, such as meat quality [2,5], population dynamics [6], and predation [7], there is a concerning lack of recent studies to verify the authenticity of roe deer meat sold in Poland. The only existing study is qualitative and dates back several years [8].

Concerns about meat product adulteration are rising globally due to potential economic fraud, violation of ethical or religious dietary restrictions, and even health risks associated with consuming undeclared species [9]. A recent inspection conducted by the Polish food inspection authority, The Inspectorate for Commercial Quality of Agricultural and Food Products (IJHARS), revealed that over 80% of inspected establishments were guilty of irregularities in declared food items. Specifically, 54% of tested popular meat-based products, such as Kebab, contained unidentified meat species [10,11]. Consequently, it is crucial to establish a reliable species-specific method for accurate quantitative detection of game meat in ready-to-eat or processed commercial products.

To address the growing concerns about food authenticity, DNA-based methods have become the preferred analytical approach for species detection in animal products due to their accuracy and reliability [12,13]. DNA contains unique species-specific information, maintaining its stability under challenging technological conditions, and setting it apart from other food components like proteins or metabolites [14]. The polymerase chain reaction (PCR)-based methods have been extensively applied for identifying meat species such as goat, chicken, turkey, pork, beef, horse, and sheep utilizing techniques like species-specific PCR, PCR with restriction fragment length polymorphisms (RFLP), multiplex PCR, real-time PCR, and digital PCR, among others [8,9,14,15,16,17,18,19,20,21].

The past two decades have witnessed significant advancements in developing reliable methods for game meat verification. These advances have mainly focused on real-time PCR methods because of the advantages of high sensitivity, specificity, and aptitude for multiplex detection and quantification [12,13]. Game meat species, including deer species (red, fallow, roe, and sika) and chamois, have been detected by real-time PCR assays [21,22,23,24,25,26,27,28,29,30,31]. Roe deer meat was also detected and quantified by a TaqMan real-time PCR assay targeting the lactoferrin gene, reaching a limit of detection (LOD) of 0.03% (*w*/*w*) and a limit of quantification (LOQ) between 0.5 and 0.125%, using serially diluted DNA extracts from a model meat mixture with 2% roe deer in pork [31]. Similarly, the same gene was targeted to detect and quantify roe deer using serially diluted DNA to achieve an LOD and LOQ of 0.1% and 0.25% of targets in non-target DNA [23,24]. Afterward, a tetraplex real-time PCR assay with MGB probes targeting nuclear genes of four deer species (roe, red, fallow, and sika) was developed, achieving an LOD and LOQ of 0.1% and 0.5% (*w*/*w*), using DNA mixtures as calibrators [25]. Despite the progress made in quantifying roe deer meat using nuclear genes and TaqMan probes, limitations remain in the design of the quantitative approaches, particularly using serially diluted DNA extracts or DNA mixtures instead of real model mixtures as calibrators to simulate food matrices. Previous studies have successfully used reference model mixtures as calibrators to estimate red deer meat in foods by real-time PCR, simulating food matrix and thermal processing, as more realistic, and at the same time, more challenging approaches [27].

This work addresses these limitations by proposing a novel and efficient roe deer species-specific quantitative real-time PCR (qPCR) method. Compared to existing methods, it targets the nuclear gene coding for the agouti signaling protein (ASIP) and proposes a calibration model based on matrix-adapted standards for the first time. For this purpose, reference model mixtures, imitating scenarios where roe deer meat is mixed with other meat types and spices and accounting for the influence of thermal processing were prepared. Furthermore, to ensure its effectiveness in real-world situations, the method was validated using blind samples and applied to commercially available game meat products. The use of a relative standard curve method with reference materials produced under market-realistic conditions increases the practicality and reliability of product quantification. Additionally, incorporating an endogenous reference gene amplified alongside the target gene allows method normalization and ensures accurate quantification by the real-time PCR system, minimizing potential variations in DNA extraction yield or degradation [9,19,20]. Therefore, this research aims to fill the existing gap by introducing a robust and specific quantification method for roe deer meat. The developed method will contribute to the integrity of the European meat market, having the potential to be applied for global food chain authentication. By ensuring accurate labeling and preventing meat product adulteration, this method can contribute to protecting consumers, upholding ethical and religious dietary practices, and promoting fair trade practices within the meat industry.

## 2. Materials and Methods

### 2.1. Samples

Fresh meat samples from roe deer, fallow deer, red deer, pork, chicken, cattle, lamb, goat, rabbit, turkey, duck, horse, guinea fowl, pigeon, pheasant, quail, and wild boar were obtained from reliable sources in markets across Poland and Portugal. Additionally, samples from twenty plant food species (wheat, soybean, corn, pine nut, garlic, rosemary, parsley, onion, chili, white pepper, coriander, bay leaf, mustard, olive, vine, sage, fennel, oregano, ginger, and sweet chili), commonly found in meat products, were included for specificity testing (Table S1, Supplementary Materials).

Commercial meat products (46) were purchased at local markets in Portugal and Poland, as well as online stores in Spain, for evaluation in the present work. These samples comprised pâtés, sausages, hams, canned meat, and dumplings, primarily labeled with deer species in different amounts, mixed with other meats and herbs/spices. The samples underwent separate mincing/homogenization in a laboratory knife grinder Grindomix GM200 (Retsch, Haan, Germany), utilizing different materials and blender containers. Prior to processing, the equipment was treated with a DNA decontamination solution.

### 2.2. Reference Mixture Preparation

The reference mixtures were made to mimic roe deer pâté using meats of roe deer, pork muscles (loin), liver, and neck, as well as various spices/condiments, including salt, onion, garlic, pepper, coriander, ginger, and rosemary. The meat samples were finely chopped into smaller portions, with the outer layers discarded. Initially, a blank mixture was prepared with pork liver, neck, and loin, in equal proportions, followed by the addition of the referred spices/condiments. Subsequently, a reference mixture with 50% roe deer was prepared with the addition of 300 g of minced roe deer meat to the same amount (300 g) of the pork blank mixture. The meats were further ground and homogenized in a kitchen robot Bimby^®^ (Vorwek, Wuppertal, Germany), where the pâtés were produced. Serial additions of blank mixture were performed to prepare the following reference mixtures 10%, 5%, 1%, 0.5%, 0.1%, 0.05%, and 0.01% (*w*/*w*) of roe deer in pork pâté. To simulate the industrial preparation of pâtés in cans or bottles, a portion of each model mixture was autoclaved at 115 °C for 30 min.

To validate the method, blind samples were independently prepared. Two sets of blind mixtures, containing 0.3%, 3%, 15%, and 30% (*w*/*w*) of roe deer in pork pâté, were produced following a similar methodology to the reference mixtures.

Reference mixtures and samples were handled and homogenized separately, using materials and containers treated with a DNA decontamination solution. The raw set of mixtures was promptly stored at −20 °C to mitigate enzymatic activity and prevent microbial growth, while the set mixtures mimicking thermal processing were immediately autoclaved at 115 °C for 30 min and later stored at −20 °C.

### 2.3. DNA Extraction

DNA from raw meats, reference and validation mixtures, and commercial samples was extracted with the NucleoSpin food kit (Macherey-Nagel, Düren, Germany) with a slight modification: adding 2 μL of RNase (2 mg/mL) after the cell lysis step. The NucleoSpin Plant II kit (Macherey-Nagel, Düren, Germany) was used to extract plant DNA according to the manufacturer’s instructions. DNA extractions were accomplished in duplicate (200 mg or 100 mg of each meat or plant sample, respectively) and stored at −20 °C.

The quality of DNA extracts was evaluated regarding purity and yield using a microplate UV spectrophotometry reader (SPECTROstar Nano, BMG Labtech, Ortenberg, Germany) and a reduced volume LVis plate accessory (BMG Labtech, Ortenberg, Germany). The DNA concentration was determined with the nucleic acid quantification protocol for double-strand DNA and absorbency data were assessed with the software MARS data analysis (BMG Labtech, Ortenberg, Germany).

### 2.4. Primers and Probe

Primers and probes were designed using publicly available resources and bioinformatic tools. The ASIP-encoding gene, which contained abundant sequences for both target and non-target species, was downloaded from the NCBI (National Center for Biotechnology Information) database for several species, including wild boar, pig, sheep, cattle, and red deer, and imported into Geneious Prime software (version 2023.2.1), where they were aligned and screened for regions with nucleotide dissimilarities among species. Primers were designed to amplify a 120-bp region of ASIP gene, tested for specificity using the PrimerBlast tool (https://www.ncbi.nlm.nih.gov/tools/primer-blast/index.cgi?GROUP_TARGET=on, accessed on 18 September 2023) and short sequences Blast (https://blast.ncbi.nlm.nih.gov/Blast.cgi, accessed on 18 September 2023) available on the NCBI resources page. The probe was designed with the Integrated DNA technologies tool, Primer Quest (https://eu.idtdna.com/PrimerQuest/Home/Index, accessed on 18 September 2023) and labeled with FAM and BHQ1 as reporter and quencher dyes, respectively. In addition, the OligoCalc software (http://biotools.nubic.northwestern.edu/OligoCalc.html, accessed on 18 September 2023) was used to verify the absence of hairpin formation, dimerization, and self-hybridization, as well as primer properties.

Universal primers targeting a conserved eukaryotic nuclear 18S rRNA gene fragment were used to assess the amplification capacity of DNA extracts. Additionally, the same primers and probe were used for real-time PCR method normalization [32].

The primers and probes were synthesized by Eurofins MWG Operon (Ebersberg, Germany), and their sequences are displayed in Table 1.

### 2.5. Qualitative PCR

PCR assays were run in a 25-µL total reaction volume containing 2 µL of DNA extract (100 ng), ultrapure water (Sigma-Aldrich, Steinheim, Germany), buffer (67 mM Tris-HCL (pH 8.8), 16 mM (NH_4_)_2_SO_4_, 0,01% Tween 20), 200 µM dNTP (Bioron, Ludwigshafen, Germany), 3.0 mM (ASIP-F/R) or 1.5 mM (18SRG-F/R) of MgCl_2_, 1 U of SuperHot Taq DNA Polymerase (Genaxxon Bioscience GmbH, Ulm, Germany), and 200 nM (ASIP-F/R) or 240 nM (18SRG-F/R) of each primer (Table 1). The amplifications were carried out in a MJ Mini thermal cycler (Bio-Rad, Hercules, CA, USA) with the following program: initial denaturation at 95 °C for 5 min; 40 or 33 cycles (ASIP-F/R or 18SRG-F/R, respectively) at 95 °C for 30 s, 65 °C for 30 s, and 72 °C for 30 s; and a final extension at 72 °C for 5 min.

The PCR amplicons were analyzed by electrophoresis in a 1.5% agarose gel containing 1× Gel Red (Biotium, CA, USA) for staining and carried out in 1× SGTB buffer (GRISP, Porto, Portugal) for 25–30 min at 140 V. A digital image was obtained with under a UV light tray Gel Doc™ EZ System (Bio-Rad Laboratories, Hercules, CA, USA) and Image Lab software version 5.2.1 (Bio-Rad Laboratories, Hercules, CA, USA).

### 2.6. Real-Time PCR

Real-time PCR assays were conducted in 20-µL reaction mixtures composed of 2 µL (100 ng) DNA extract, 160 nM of each probe (ASIP-P or 18SRG-P), 240 or 280 nM of each primer (ASIP-F/R or 18SRG-F/R) (Table 1), and 1× SsoFast Probes Supermix (Bio-Rad Laboratories, Hercules, CA, USA). Each target gene (ASIP and 18S rRNA) was amplified in parallel reactions and ran simultaneously in the fluorometric thermal cycler CFX96 real-time PCR detection system (Bio-Rad Laboratories, Hercules, CA, USA), following the temperature conditions of 95 °C for 5 min, 50 cycles at 95 °C for 10 s, and 65 °C for 45 s, with a fluorescence signal collection at the end of each cycle. The analysis output was processed using the Bio-Rad CFX Manager 3.1 (Bio-Rad Laboratories, Hercules, CA, USA). Real-time PCR assays were conducted in three independent runs, each with *n* = 4 replicates.

The Minimum Information for Publication of Quantitative Real-Time PCR Experiments (MIQE) guidelines [33] were meticulously adhered to, complemented with the criteria established by the European Network of GMO Laboratories (ENGL) for real-time PCR method development and validation [34]. The acceptable criteria outlined by both MIQE and ENGL, including specificity, a dynamic range covering four orders of magnitude, a coefficient of regression value ≥ 0.98, a slope ranging between −3.6 and −3.1, and a PCR efficiency between 90% to 110%, were ensured throughout the experiment. The limit of detection (LOD) was considered as the lowest quantity of the analyte in a sample that should be detected with a confidence level of 95%, safeguarding no more than 5% of false-negative results. The limit of quantification (LOQ) was established as identical to or lesser than the lowest quantity within the dynamic range [33,34].

### 2.7. Statistical Analysis

GraphPad Prism version 8.0.2 software (GraphPad Software, San Diego, CA, USA) was used for statistical analysis. The Shapiro–Wilk test was used to evaluate the normality of the data, meaning that data following normal distribution were analyzed by an unpaired *t*-test. In contrast, the non-parametric Mann–Whitney test was used for testing data following a non-normal distribution. The significance of differences was determined regarding the comparison of Cq values of raw and processed pâtés at the same concentration level for the normalized calibration method. Significant statistical differences were considered when *p* < 0.05.

## 3. Results and Discussions

### 3.1. Method Optimization and Specificity Evaluation

A critical aspect in method development lies in the design of species-specific primers and probes. In this work, the focus was on designing and optimizing novel species-specific primers targeting the ASIP-encoding gene, responsible for the pigmentation regulation in roe deer. This gene was selected considering the availability of sequences in the NCBI database. Moreover, it is a nuclear gene, thus having fixed copy number regardless of tissue and cell type and being more suitable for quantitative analysis, in opposition to mitochondrial DNA markers that have variable copy numbers. To enhance specificity and uniqueness, several measures were applied. Firstly, a short product length of 120 bp was ensured to restrict the hybridization site, minimizing non-specific binding. Secondly, PCR temperature conditions were methodically optimized through repeated assays to identify the optimal parameters providing the highest product yield while avoiding cross-amplification.

The addition of TaqMan probes provided extra specificity by targeting a specific region within the amplified DNA sequence [34,35]. Additionally, the incorporation of the 18SRG primers [32] as a universal marker not only confirmed the presence of amplifiable DNA in all tested samples but it also allowed the normalization of the real-time PCR system by adopting the ∆Cq (cycle quantification difference) approach for quantification [9,19,20]. This method is based on the construction of a calibration curve that involves measuring two specific DNA sequences, carefully selected with comparable efficiencies, the reference universal eukaryotic gene (nuclear 18S rRNA) and the species-specific target (ASIP), using the following expression:

∆Cq = Cq_ASIP_ − Cq_euk_

where Cq_ASIP_ and Cq_euk_ represent the cycles of quantification for roe and eukaryotic systems, respectively.

Another significant component of the method development was obtaining high-quality DNA extracts with appropriate yield and high purity levels of 1.8 (A260/A280) and 2.3 (A260/A230) for roe deer. Generally, high-quality DNA was isolated for all species. The extracted DNA was used for specificity and sensitivity testing alongside developed and optimized primers and probes.

Prior to real-time PCR method development, the attainment of high-quality DNA extract with appropriate yield and purity level was assured for all mixtures and tested species. PCR optimization was crucial for achieving a high level of method specificity and sensitivity. Temperature gradients were tested with three annealing temperatures (60 °C, 62 °C, and 65 °C) and 65 °C was identified as the optimal temperature for enhancing target amplification while minimizing non-specific binding. Furthermore, slight adjustments to MgCl_2_ concentration, cycle number, and stringent contamination prevention protocols were also essential for obtaining a clean specificity profile.

The method development progressed to the establishment of calibrators for quantitative analysis with two sets of reference model mixtures (raw and autoclaved pâtés) prepared by adding the target species from 50% to 0.01% of roe deer to pork pâté. Blind samples with varying proportions of roe deer meat was also produced to validate the established method before application to commercial samples.

To ensure method accuracy and avoid false positive results, primer specificity was assessed in silico against a broad range of potential cross-reactivity sources using tools such as NCBI Primer-Blast and Short Sequence Blast. Since no cross-reactivity was detected, the analysis shifted to in vitro testing of 16 animal species, namely pig and wild boar, other cervids (red deer and fallow deer), birds and poultry, cattle, rabbit, and others, as well as 20 plant species commonly found in processed meat products, including spices and crops such as soybean, wheat, corn, and herbs. The results show no reactivity with non-target species, except for red deer (*Cervus elaphus*) and fallow deer (*Dama dama*), producing the expected 120-bp fragment (Table S1, Supplementary Materials). The amplification with 18SRG primers confirmed the absence of any false negative result (Table S1, Supplementary Materials). Additionally, a hydrolysis probe was designed for real-time PCR to enhance the specificity of the assay for roe deer, avoiding the reactivity with red deer and fallow deer. Figure S1 (Supplementary Materials) shows the real-time PCR amplification curves using a TaqMan probe targeting the ASIP gene and DNA extracts of roe deer, red deer, and fallow deer, thus confirming the specificity for the roe deer.

### 3.2. Sensitivity

**Figure 1:**
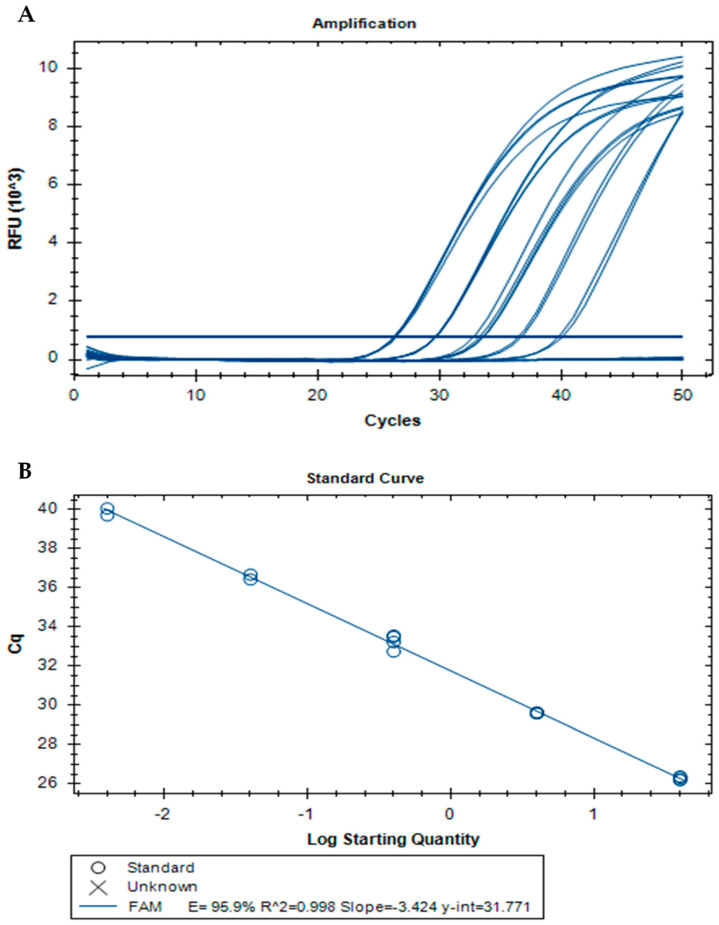
Amplification (**A**) and standard (**B**) curves obtained by real-time PCR with a TaqMan probe targeting the agouti signaling protein-encoding gene (roe deer DNA 40–0.004 ng, *n* = 4 replicates).

The absolute sensitivity was determined using a 10-fold serial dilution of a roe deer DNA extract, ranging from 40 ng to 0.004 ng. The serially diluted DNA was first assayed by qualitative PCR targeting the ASIP gene of roe deer, producing the expected 120-bp fragment down to 0.004 ng of DNA (Figure S2, Supplementary Materials). Afterward, the serially diluted roe deer DNA was amplified by real-time PCR with ASIP primers and a TaqMan probe. Figure 1 shows one example of a real-time PCR run, with the amplification curves (Figure 1A) and respective calibration curve (Figure 1B), with a dynamic range covering 4 orders of magnitude. As the level of 0.004 ng was not amplified in all replicates (*n* = 12), the absolute LOD was established as 0.04 ng to ensure a detection level of confidence above 95%. The absolute LOQ was equal to the LOD as it was within the calibration curve linearity [33]. The assay parameters of PCR efficiency (95.5%), slope (−3.424), and correlation coefficient (*R*^2^) of 0.998 complied with the acceptable criteria for real-time PCR assays (Figure 1B) [33,34].

The obtained LOD (0.02 ng/µL) was slightly higher than the previously reached value for red deer targeting the troponin I gene (0.005 ng/µL) [27] and lower than that of other reports for roe deer targeting the lactoferrin gene (0.08 ng/µL) [31]. Therefore, the obtained LOD agrees with other reports targeting nuclear genes. In opposition, much lower sensitivities for deer species and other mammal or avian species were achieved by targeting mitochondrial genes, namely the ND5 gene for red deer (0.25–0.5 pg) [30] and the *cytb* gene for horse (0.1 pg) [9], pork (0.01 pg) [19], pigeon (0.1 pg), and chicken (0.1 pg) [18]. This is justified by the fact that mitochondrial genes are multicopy markers that allow much-increased sensitivity, but on the other hand, this is a drawback for accurate species quantification.

The relative LOD was evaluated using reference mixtures containing the target DNA (roe deer) at various proportions (50% to 0.01%, *w*/*w*) within a complex matrix simulating commercially available pâté (both raw and autoclaved). Both real-time PCR systems effectively detected the target species down to a concentration of 0.05% (*w*/*w*) in raw and heat-treated mixtures (Table 2). The LOD was established through three independent assays with four replicates each, ensuring less than 5% false-negative results.

The relative LOD obtained in this study is lower than other previously reported for roe deer. An LOD within 0.32–0.64% (*w*/*w*) for roe deer was reported using a multiplex real-time PCR and DNA dilutions [21], while other authors achieved a relative LOD of 0.1% (*w*/*w*) also using serially diluted DNA or DNA mixtures [25]. Although an LOD of 0.03% (*w*/*w*) was obtained in another study [31], such a level was reached by analyzing serially diluted DNA extracts. Therefore, the present study reports, for the first time, a relative LOD accounting for the influence of food matrix and food processing, which is only comparable with the previously obtained LOD for red deer (0.5%, *w*/*w*) using reference model mixtures [27].

### 3.3. Normalized Calibration Curve

**Figure 2:**
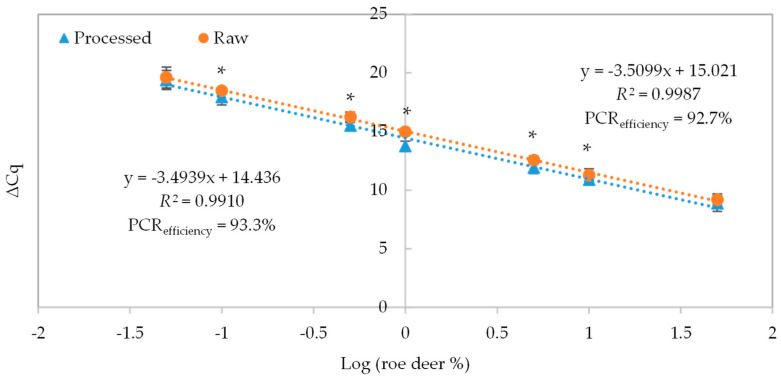
Normalized calibration curves achieved by TaqMan real-time PCR targeting the agouti signaling protein-encoding gene using the ∆Cq method (∆Cq = Cq _ASIP_ − Cq _euk_) and reference mixtures of 50% to 0.05% (*w*/*w*) of roe deer meat in simulated pâté, raw (●), and thermally processed forms (▲) (*n* = 12 replicates). * Mean statistical differences (*p* < 0.05) following an unpaired *t*-test or Mann–Whitney test.

In the present work, a quantitative method based on reference model mixtures was proposed to determine the amount of roe deer in meat products. The method used two sets of DNA extracts of reference model mixtures within 0.01–50% (*w*/*w*) of roe deer in simulated pâté, as raw and thermally processed. The real-time PCR amplification results targeting the ASIP gene of roe deer are displayed in Table 2, exhibiting very good linearity within 0.05–50% for both raw (*R*^2^ = 0.9975) and thermally processed mixtures (*R*^2^ = 0.9964). Regarding the slope and PCR efficiency of calibration curves, the obtained values are within the criteria of acceptance for the raw pâté calibrators (slope = −3.5834 and efficiency = 90.1%), but slightly below the recommended level for the autoclaved pâté calibrators (slope = −3.7120 and efficiency = 86.0%). Consequently, as an attempt to improve the real-time PCR quantitative performance, the parallel amplification of an endogenous reference gene was performed to normalize the calibration models (Table 2). This approach considers eventual amplification differences owing to variable DNA recovery, purity, and integrity because of food matrix and processing [9,19,20]. Figure 2 presents the normalized calibration curves using the ΔCq values of reference mixtures of 50% to 0.05% (*w*/*w*) of roe deer meat in simulated pâté, raw and after thermally processed. As verified, the obtained curve parameters met the acceptance criteria regarding the correlation coefficient (*R*^2^ > 0.99), slope (−3.5099 and −3.4939 for raw and autoclaved mixtures, respectively) and PCR efficiency slope (92.7% and 93.3% for raw and autoclaved mixtures, respectively). Therefore, normalization improved considerably the performance of both calibration models as inferred by their enhanced PCR efficiency. Both calibration plots are very similar, particularly regarding slope, exhibiting a small intercept deviation of nearly 0.6 cycles (Figure 2). This difference was quite reduced with method normalization, shifting from 1.3 to 0.6 cycles (Table 2), indicating that this process effectively attenuated the influence of thermal processing. Nonetheless, statistical differences were observed between raw and processed model mixtures for all levels of amplification, except for the 5% and 0.05% of roe deer in pâté (Figure 2). Differences between raw and severely processed matrices, such as autoclaved meats, are expected to occur due to the loss of DNA integrity, with consequent delayed amplification [9,19]. Therefore, the construction of a calibration curve accounting for the influence of autoclaving is crucial in the case of pâté samples. However, the same is not expected to occur for cured or mildly thermally treated products owing to their stabilized enzymatic activity because of the low water activity or thermal treatment, respectively. This was previously observed for the quantification of soybean material in meat products, where the normalized calibration curves for raw and mildly processed mixtures (66–68 °C for 5 h) were practically overlapping [32]. The feasibility of method normalization using the ΔCq method was previously demonstrated in the quantification of other meat species such pork [19,20] and horse [9] in food products, as well as plant species such as soybean [34].

The LOQ, established as the lowest amount of the analyte that can be reliably measured, was established in this study at 0.05% (*w*/*w*) of roe deer meat in pork pâté for both raw and heat-treated mixtures. This study achieved a significant improvement compared to previous research attaining LOQ values between 0.125% and 0.5% (*w*/*w*) for roe deer [23,25,31]. Furthermore, an interlaboratory ring trial involving 14 European laboratories employed meat mixtures ranging from 2% to 49.4% for roe deer content [26], highlighting the widest quantitative range of the proposed method targeting roe deer herein.

The present study reports, for the first time, a normalized calibration curve prepared with matrix-adapted standards for the quantification of roe deer, accounting for the influence of food matrix and food processing, which is only comparable with the previously obtained calibration curve targeting red deer [27].

### 3.4. Validation

Method validation is required to establish the reliability, consistency, and accuracy of the developed novel method, confirming its fitness for quantification and its easy adoption in different laboratories [36]. Accordingly, four blind sample pairs were prepared with varying proportions (30%, 15%, 3%, and 0.3%, *w*/*w*) of roe deer meat incorporated into both raw and autoclaved pork pâté and used to assess the effectiveness of the quantitative real-time PCR assay. The roe deer contents of raw and thermally treated blind samples were estimated using the respective normalized calibration models of Figure 2 and compared with the actual values displayed in Table 3. Key parameters such as coefficient of variation (CV) and bias were considered in this evaluation to assess the method’s precision and trueness, respectively. The CV data, expressing the relative standard deviation of the results obtained under repetitive conditions, were within 8.1–23.0% and 7.0–17.5% for raw and processed samples, respectively. The bias data, expressing the proximity between the actual and estimated roe deer contents, varied from −21.9 to 1.9% and from −12.1 to 13.6%, for raw and processed samples, respectively. Therefore, both CV and bias data meet the acceptance criteria (CV ≤ 25% and bias ±25%) for quantification by real-time PCR assays [34].

### 3.5. Applicability of the Method to Commercial Products

In total, 46 marketed meat products originating from four European countries, Poland (28 samples), Spain (8 samples), Portugal (6 samples), and France (4 samples), were tested to verify labeling compliance. These products encompassed various types of processed foods, including loin, canned meat, ham, terrine, pâté, salami, and sausages. Product labeling indicated the following meat species: 28 red deer, 14 roe deer, 2 fallow deer, and the remaining 2 wild boar or pig meat (Table 4). This diverse selection of products and game species served three purposes: (i) validation—final method validation through its application to commercial deer-containing products; (ii) labeling compliance—to assess the accuracy of manufacturer claims and ensure product labeling reflects the actual ingredients; and (iii) adulteration survey—to estimate the level of mislabeling or adulterations in roe deer meat products in several European countries. The results of the roe deer determination in the 46 commercial samples are summarized in Table 4, complemented with the red deer contents obtained in our previous study [27].

To proceed with roe deer detection and quantification, the commercial products were split into two groups based on their production methods: uncooked/mildly cooked up to 71 °C/cured samples (sausages #1–#24) and thermally treated samples (pâtés #25–#46). The appropriate calibration models, for raw and autoclaved mixtures, respectively, were then employed to each group. All products underwent an initial test to detect amplifiable DNA by qualitative PCR targeting of a universal marker of the nuclear 18S rRNA gene and by discarding any false negative result. Subsequently, the roe deer-specific ASIP primers were used to qualitatively determine the presence of roe deer DNA (Table 4). As summarized in Table 4, of the 14 products labeled as containing roe deer meat, the target species was detected by both qualitative PCR and real-time PCR in 9 of them. The analysis of those samples, declaring roe deer contents of 7.2% to 100% (*w*/*w*), revealed a significant discrepancy between estimated and labeled values for roe deer. All of them contained a substantially lower amount of roe deer than what was declared, with estimated values falling between 0.07% and 19.23% (*w*/*w*) (Figure S3, Supplementary Materials). Sample #34, with the least significant difference between declared and quantified roe deer content, only contained half the labeled amount. Sample #15 had the lowest quantified value at 0.07%, and roe deer DNA was undetectable or below the detection limit in samples #11–#14, and #37. Other samples (#16, #31-#33, #35, #42, and #44) suffered high reductions within 70–90%. These results suggest that all roe deer labeled samples were adulterated by total and substantial substitution in 6 and 8 samples, respectively. This is a serious issue of product mislabeling for consumers, particularly concerning a product that typically commands a higher price compared to regular meat products.

For samples labeled as containing other game species, namely red deer (28), fallow deer (2), and pork/wild boar (2), roe deer DNA was quantified in 8 red deer samples (#1, #2, #7, #17, #18, #25, #26, and #29) and one fallow deer/game product (#19). In samples #1, #2, and #29, the estimated trace levels of roe deer (0.10–0.36%) might be attributed to contamination rather than adulteration, though sample #29 also declares roe deer. The remaining samples showed contents within 1.40–6.53% of roe deer. Interestingly, all these samples, except for sample #1 with 0.49% of red deer, also exhibited high or considerable contents of red deer. This result suggests that undeclared roe deer addition was performed to increase the proportion of deer species rather than replacing red deer.

The complementary analysis of both roe deer and red deer contents highlights that in samples #1, #11, and #30, the deer species were probably fully substituted with pork. The contents of samples #12–#15 and #37 suggest full substitution of roe deer with red deer, while samples #16 and #35 indicate partial replacement of roe deer with red deer. Fallow deer meat was replaced with red deer/roe deer and red deer in samples #19 and #36, respectively. Samples #20–#23, #25–#26, #30–#34, #38–#40, and #42–#44 indicate reduced proportions of red or roe deer, suggesting its substitution with pork. Moreover, undeclared roe deer was added in samples #2, #7, #17, and #18. Therefore, the results of roe deer detection and quantification improved the previous survey on red deer in game meat products that indicated an adulteration/mislabeling level of 48% [27], which was further complemented and increased to 61%. This increase was also due to considering the partial reduction in labeled species in the present analysis.

Summarizing the analysis of all 46 products by country of origin and type of mislabeling/adulteration, the data revealed that 100% of samples from Portugal had their red deer content reduced, suggesting its partial substitution with pork. The same type of adulteration was observed in 50% of samples from France. In 87.5% of samples from Spain, two types of mislabeling/adulterations were noted, namely the addition of undeclared roe deer and species substitution of fallow deer with other deer species and red deer with pork. The samples of Poland accounted for the most representative group, but they were also diverse regarding the identified mislabeling/adulterations (57%), which were due to the absence of deer species, reduced deer content, substitution of roe deer with red deer, and undeclared addition of roe deer.

The results underscore the importance of routine testing for meat products, particularly to identify adulteration through addition, substitution, and mislabeling. The results showed significant mislabeling across various game meat products. Roe deer meat was detected at trace levels in products labeled as containing other species, also suggesting contamination within the game industry. Furthermore, undeclared roe deer content in some products highlights major discrepancies in labeling, raising concerns about transparency and potential consumer deception. The reduced amount of the labeled roe deer species as well as the presence of undeclared red deer in roe deer-labeled products can indicate economically motivated adulteration practices. However, it is important to emphasize that these findings may not necessarily reflect the general food safety practices within these countries, but rather the specific samples included in this study. The mislabeling detected is likely caused by species misidentification due to their close evolutionary relationship belonging to the Cervidae family and, therefore, non-malicious mistakes made in the addition of meat from deer species by less skilled workers. The second highly probable reason may be differences in the price of deer meat sold by hunting districts and the fact that red deer meat is, in general, half the price of roe deer. Finally, the third reason may be the difference in the availability of fresh game meat throughout the year, i.e., roe deer meat is much more expensive but available for processing for more months of the year compared to red deer, approximately nine months versus six months.

Overall, the high prevalence of mislabeled or adulterated products underlines the need for stricter regulations and more rigorous monitoring to ensure consumer trust and adherence to labeling standards. The present results highlight the need to extend the study to a wider range of game meat products in EU-consuming countries, such as Italy, Germany, Czechia, the United Kingdom, and Sweden. The diversity of products should also be enlarged, including traditional products like stew/goulash and dumplings, as well as wild boar products considering their high relevance.

## 4. Conclusions

This research successfully addressed the issue of roe deer meat adulteration by developing a novel real-time quantitative PCR system targeting the roe deer ASIP-encoding gene. The method utilizes well-designed reference model mixtures that accurately reflect commercially available products. Two quantitative real-time PCR systems, one suitable for raw/mildly treated products and another for severely processed (autoclaving) meat products, were successfully developed using the normalized ΔCq method. Both systems achieved absolute and relative LOD and LOQ of 0.04 ng of DNA and 0.05% (*w*/*w*) of roe deer in simulated pâté, respectively. Following validation with eight blind samples, which highlighted the precision and trueness of the approach, the method was applied to commercial products. The analysis of 46 game meat samples from Poland, Portugal, Spain, and France revealed a high level of discrepancies between manufacturers’ claims and the actual presence of roe deer in some products. All the 14-roe deer labeled samples were mislabeled/adulterated by total or partial species substitution. A global analysis of all samples, combining the results of our previous study surveying red deer species, identified 61% of mislabeled/adulterated samples due to the absence of deer species, reduced deer content, substitution of roe deer with red deer, substitution of fallow deer with other deer species and red deer with pork, and undeclared addition of roe deer. This highlights the effectiveness of the method in detecting/quantifying meat adulterations.

Therefore, the developed real-time PCR assay proved to be an effective tool for detecting and quantifying roe deer, demonstrating its potential as a reliable method for food authenticity testing. This method significantly contributes to the existing knowledge on roe deer meat authentication and demonstrates its usefulness as a tool for food inspection to ensure effective labeling compliance. Consequently, it will contribute to valorizing the game meat products, while promoting consumer trust and transparency, as well as fair trade among producers.

## Figures and Tables

**Table 1:** Primers and probes used in this study.

Species	Target Gene	Primer	Sequence (5′-3′)	Amplicon	NCBI Accession/Reference
*Capreolus capreolus*	Agouti signaling protein	ASIP-F	GAGTCCATTTTCCAGGGCCG	120 bp	NC_057828.1
		ASIP-R	GGGACTTAGGCCTGCAGTTT		
		ASIP-P	FAM-ACTCTCTGCTCTCTGTCTCTGGCT-BHQ1		
Eukaryotes	18S rRNA	18SRG-F	CTGCCCTATCAACTTTCGATGGTA	113 bp	[32]
		18SRG-R	TTGGATGTGGTAGCCGTTTCTCA		
		18SRG-P	FAM-ACGGGTAACGGGGAATCAGGGTT CGATT-BHQ1		

**Table 2:** Real-time PCR results using reference mixtures of roe deer in simulated pâté (raw and autoclaved) targeting a reference gene (18S rRNA) and an agouti signaling protein (ASIP)-encoding gene of roe deer.

Model Mixtures Containing Roe Deer (%, *w*/*w*)	Raw Pâté Mixtures			Autoclaved Pâté Mixtures		
	Cq ± SD ^a^ (18S rRNA)	Cq ± SD (ASIP)	Replicates (Positive/ Total)	Cq ± SD (18S rRNA)	Cq ± SD (ASIP)	Replicates (Positive/ Total)
50	16.46 ± 0.46	25.67 ± 0.10	(12/12)	17.74 ± 0.48	26.81 ± 0.42	(12/12)
10	16.49 ± 0.39	27.77 ± 0.08	(12/12)	18.34 ± 0.30	29.24 ± 0.38	(12/12)
5	16.74 ± 0.24	29.30 ± 0.06	(12/12)	18.20 ± 0.49	30.37 ± 0.47	(12/12)
1	16.97 ± 0.25	31.94 ± 0.17	(12/12)	19.09 ± 0.45	32.90 ± 0.51	(12/12)
0.5	16.62 ± 0.33	32.88 ± 0.21	(12/12)	19.08 ± 0.51	34.50 ± 0.47	(12/12)
0.1	16.81 ± 0.31	35.28 ± 0.21	(12/12)	18.40 ± 0.35	36.36 ± 0.53	(12/12)
0.05	16.60 ± 0.33	36.20 ± 0.37	(12/12)	18.48 ± 0.44	38.09 ± 1.10	(12/12)
0.01	NA	NA	(0/12)	NA	NA	(0/12)
*R* ^2^		0.9975			0.9964	
Slope		−3.5834			−3.7120	
Intercept		31.698			33.031	
PCR_efficiency_ (%)		90.1			86.0	

^a^ Mean quantification cycle (Cq) ± standard deviation (SD).

**Table 3:** Validation data determined by quantitative real-time PCR applied to blind mixtures.

Blind Samples	Actual Value (%)	Estimated Value (%) ^a^	SD ^b^	CV (%) ^c^	Bias (%) ^d^
Raw mixtures					
A	30	30.57	5.93	19.4	1.9
B	15	13.44	1.08	8.1	−11.6
C	3	2.46	0.22	8.8	−21.9
D	0.3	0.29	0.07	23.0	−2.2
Processed mixtures					
E	30	34.29	5.99	17.5	12.5
F	15	13.88	2.30	16.5	−8.0
G	3	3.47	0.24	7.0	13.6
H	0.3	0.27	0.05	17.9	−12.1

^a^ Mean values of replicate assays (*n* = 12). ^b^ SD—standard deviation. ^c^ CV—coefficient of variation. ^d^ Bias = ((mean estimated value − true value)/true value × 100).

**Table 4:** Analyzed of commercial game meat products by qualitative PCR and quantitative real-time PCR using the normalized ΔCq method targeting a reference gene (18S rRNA) and the agouti signaling protein (ASIP) gene of roe deer.

Code	Sample Description	Relevant Label Information	Origin	Qualitative PCR ^a^		Real-Time PCR				Estimated Red Deer Content (Mean ± SD, % *w*/*w*) [29]
				18SRG-F/18SRG-R	ASIP-F/ASIP-R	18S rRNA (Cq ± SD) ^b^	ASIP (Cq ± SD) ^b^	Estimated Roe Deer Content (mean ± SD, % *w*/*w*)	CV ^c^ (%)	
Uncooked/cured										
#1	Salami with red deer	10% red deer meat	Poland	+	+	18.08 ± 0.11	37.21 ± 0.43	0.10 ± 0.00	3.61	0.49 ± 0.05
#2	Red deer and pork kabanos	51% red deer meat	Poland	+	+	16.54 ± 0.26	33.69 ± 0.42	0.36 ± 0.02	5.53	>50
#3	Dry mysliwska sausage with red deer	25% red deer meat	Poland	+	−					20.37 ± 1.61
#4	Red deer sausage	70% red deer meat	Poland	+	−					>50
#5	Red deer sausage Wild grill	30.6% red deer meat	Poland	+	+	15.53 ± 0.09	35.99 ± 0.69	< LOD		>50
#6	Juniper sausage	>50% red deer meat	Poland	+	−					>50
#7	‘Swojska’ sausage	<50% red deer meat	Poland	+	+	15.73 ± 0.17	30.04 ± 0.16	2.18 ± 0.25	11.54	>50
#8	Red deer salami	100% red deer meat	Poland	+	−					>50
#9	Red deer ‘kindziuk’	70% red deer meat	Poland	+	−					>50
#10	Tender red deer sausage	35.5% red deer meat	Poland	+	−					>50
#11	Roe deer kabanos	100% roe deer meat	Poland	+	−					−
#12	Roe deer sausage	70% roe deer meat	Poland	+	+	16.46 ± 0.17	37.91 ± 1.65	< LOD		19.85 ± 0.98
#13	Roe deer sausage	>50% roe deer meat	Poland	+	−					44.55 ± 3.64
#14	Roe deer sausage	<50% roe deer meat	Poland	+	-					>50
#15	Roe deer kabanos	100% roe deer meat	Poland	+	+/-	16.62 ± 0.31	36.20 ± 0.82	0.07 ± 0.01	13.15	>50
#16	Roe deer sausage	<50% roe deer meat	Poland	+	+	16.32 ± 0.22	29.53 ± 0.21	4.29 ± 0.27	6.23	>50
#17	Red deer sausage	60% red deer meat	Spain	+	+	17.48 ± 0.15	30.07 ± 0.40	6.53 ± 0.65	9.92	>50
#18	Red deer sausage	60% red deer meat	Spain	+	+	16.92 ± 0.12	31.98 ± 0.49	1.40 ± 0.35	24.72	>50
#19	Spicy fallow deer sausage	60% game meat	Spain	+	+	17.16 ± 0.26	30.11 ± 0.41	5.17 ± 0.31	6.06	>50
#20	Red deer sausage	60% red deer meat	Portugal	+	−					3.85 ± 0.20
#21	Red deer sausage with fine herbs	60% red deer meat	Portugal	+	−					18.68 ± 2.22
#22	Red deer sausage with sesame seeds	60% red deer meat	Portugal	+	−					29.73 ± 0.91
#23	Red deer sausage with pepper	60% red deer meat	Portugal	+	−					7.52 ± 0.42
#24	Red deer loin	100% red deer meat	Spain	+	−					>50
Thermally treated										
#25	Baked red deer polish pâté	70% red deer meat	Poland	+	+	17.50 ± 0.32	30.31 ± 0.59	3.81 ± 0.14	3.58	20.48 ± 0.15
#26	Red deer polish pâté (canned)	70% red deer meat	Poland	+	+	17.15 ± 0.10	31.01 ± 0.42	2.50 ± 0.43	17.24	13.84 ± 1.34
#27	Red deer sausage (canned)	56% red deer meat	Poland	+	−					>50
#28	Baked red deer polish pâté	36.6% red deer meat	Poland	+	−					>50
#29	Hunter’s polish pâté (canned; 3 types of meat: red deer, wild boar, roe deer)	>33% red deer meat	Poland	+	+	18.34 ± 0.24	37.27 ± 0.72	0.11 ± 0.01	12.15	>50
#30	Red deer ham	100% red deer meat	Poland	+	−					−
#31	Baked roe deer polish pâté	70% roe deer meat	Poland	+	+	17.67 ± 0.28	28.37 ± 0.44	19.23 ± 1.87	9.71	−
#32	Roe deer polish pâté (canned)	70% roe deer meat	Poland	+	+	17.74 ± 0.30	29.19 ± 0.46	12.11 ± 0.44	3.63	−
#33	Roe deer sausage (canned)	55% roe deer meat	Poland	+	+	18.17 ± 0.40	29.30 ± 0.35	14.95 ± 1.79	11.98	−
#34	Baked polish pork pâté with roe deer and Armagnac	20% roe deer meat	Poland	+	+	16.34 ± 0.17	27.33 ± 0.17	11.18 ± 1.23	11.04	−
#35	Baked roe deer polish pâté (baked)	51% roe deer meat	Poland	+	+	17.23 ± 0.18	28.15 ± 0.26	11.80 ± 0.08	0.71	7.21 ± 1.76
#36	Fallow deer pâté	25% fallow deer meat	Spain	+	−					15.96 ± 3.00
#37	Roe deer pâté with port wine	7.2% roe deer meat	Spain	+	−					18.95 ± 1.03
#38	Red deer pâté with boletus	20% red deer meat	Spain	+	−					8.69 ± 0.44
#39	Red deer pâté	25% red deer meat	Portugal	+	−					0.87 ± 0.21
#40	Red deer pâté with extra virgin olive oil	11.62% red deer meat	Spain	+	−					6.68 ± 1.28
#41	Wild boar terrine with cognac	21% wild boar meat	France	+	−					−
#42	Roe deer terrine with cognac	21% roe deer meat	France	+	+	16.15 ± 0.41	28.12 ± 0.24	5.86 ± 1.01	17.32	−
#43	Red deer terrine with cognac	21% red deer meat	France	+	−					22.23 ± 1.33
#44	Roe deer terrine with juniper berries	21% roe deer meat	France	+	+	19.30 ± 0.31	30.97 ± 0.38	7.12 ± 0.97	13.60	−
#45	Red deer pâté	30.52% red deer meat	Portugal	+	−					7.47 ± 0.8
#46	Pork kabanus	Pork meat	Poland	+	−					−

^a^ (+), positive amplification, (−), no detected amplification. ^b^ Mean quantification cycles (Cq) of replicate assays (*n* = 8) ± standard deviation (SD). ^c^ CV—coefficient of variation.

## Data Availability

The original contributions presented in the study are included in the article/Supplementary Materials, further inquiries can be directed to the corresponding author.

## Supplementary Materials

The following supporting information can be downloaded at https://www.mdpi.com/article/10.3390/foods13233728/s1: Figure S1: Amplification curves obtained by real-time PCR with a TaqMan probe targeting the ASIP gene using DNA extracts of roe deer, red deer, and fallow deer at the same concentration (50 ng/µL); Figure S2: Agarose gel electrophoresis of PCR products targeting the agouti signaling protein (ASIP) gene of roe deer using serially diluted roe deer DNA. Legend: M, 100 bp DNA Ladder; lane 1, 40 ng; lane 2, 4 ng; lane 3, 0.4 ng; lane 4, 0.04 ng; lane 5, 0.004 ng; NC, negative control; Figure S3: Comparison of declared versus estimated roe deer contents by real-time PCR; Table S1: Results of qualitative PCR targeting a universal eukaryotic region of the 18S rRNA gene and the agouti signaling protein (ASIP) gene of roe deer using several relevant animal and plant species for cross-reactivity testing.

## References

[1] Animalia Roe Deer. https://animalia.bio/roe-deer#:~:text=According%20to%20the%20IUCN%20Red%20List%2C%20the%20central,of%20this%20species%20is%20around%2015%20million%20individuals.

[2] Milczarek A., Janocha A., Niedziałek G., Zowczak-Romanowicz M., Horoszewicz E., Piotrowski S. (2021). Health-promoting properties of the wild-harvested meat of roe deer *(Capreolus capreolus* L.) and red deer (*Cervus elaphus* L.). Animals.

[3] Burbaite L., Csányi S. (2009). Roe deer population and harvest changes in Europe. Est. J. Ecol..

[4] FACE Report (2020). The Economics of Hunting in Europe: Towards a Conceptual Framework. https://face.eu/sites/default/files/attachments/framework_for_assessing_the_economics_of_hunting_final_.en_.pdf.

[5] Kasałka-Czarna N., Biegańska-Marecik R., Proch J., Orłowska A., Montowska M. (2023). Effect of dry, vacuum, and modified atmosphere ageing on physicochemical properties of roe deer meat. Pol. J. Food Nutr. Sci..

[6] OrŁowska L., Rembacz W. (2016). Population dynamics and structure of roe deer (*Capreolus capreolus*) inhabiting small-size forests in north-western Poland. Folia Zool..

[7] Melis C., Jędrzejewska B., Apollonio M., Bartoń K.A., Jędrzejewski W., Linnell J.D.C., Kojola I., Kusak J., Adamic M., Ciuti S. (2009). Predation has a greater impact in less productive environments: Variation in roe deer, *Capreolus capreolus*, population density across Europe. Glob. Ecol. Biogeogr..

[8] Rak L., Knapik K., Bania J., Sujkowski J., Gadzinowski A. (2014). Detection of roe deer, red deer, and hare meat in raw materials and processed products available in Poland. Eur. Food Res. Technol..

[9] Meira L., Costa J., Villa C., Ramos F., Oliveira M.B.P.P., Mafra I. (2017). EvaGreen real-time PCR to determine horse meat adulteration in processed foods. LWT-Food Sci. Technol..

[10] Szyłak A., Kostrzewa W., Bania J., Tabiś A. (2023). Do you know what you eat? Kebab adulteration in Poland. Foods.

[11] NFP Irregularities Found in over 80% of Kebab Shops Inspected in Poland. https://notesfrompoland.com/2023/07/26/irregularities-found-in-over-80-of-kebab-shops-inspected-in-poland/#:~:text=Irregularities%20found%20in%20over%2080%25%20of%20kebab%20shops%20inspected%20in%20Poland,-Jul%2026%2C%202023&text=An%20inspection%20of%20food%20establishments,meats%20different%20from%20those%20advertised.

[12] Amaral J., Meira L., Oliveira M.B.P.P., Mafra I., Downey G. (2016). 14—Advances in authenticity testing for meat speciation. Advances in Food Authenticity Testing.

[13] Adenuga B.M., Montowska M. (2023). A systematic review of DNA-based methods in authentication of game and less common meat species. Compr. Rev. Food Sci. Food Saf..

[14] Ramos S.C., Center P.C., Balagan E., Dimalanta F. (2016). Molecular evaluation of pork, beef and poultry meat sold in Nueva Ecija, Philippines for the presence of horse (*Equus caballus*) and rat (*Rattus rattus*) DNA using polymerase chain reaction assay. Philipp. J. Vet. Med..

[15] Uddin S.M.K., Hossain M.A.M., Chowdhury Z.Z., Johan M.R. (2021). Detection and discrimination of seven highly consumed meat species simultaneously in food products using heptaplex PCR-RFLP assay. J. Food Compos. Anal..

[16] Fajardo V., González I., López-Calleja I., Martín I., Hernández P.E., García T., Martín R. (2006). PCR-RFLP authentication of meats from red deer (*Cervus elaphus*), fallow deer (*Dama dama*), roe deer (*Capreolus capreolus*), cattle (*Bos taurus*), sheep (*Ovis aries*), and goat (*Capra hircus*). J. Agric. Food Chem..

[17] Uddin S.M.K., Hossain M.A.M., Chowdhury Z.Z., Johan M.R.B. (2021). Short targeting multiplex PCR assay to detect and discriminate beef, buffalo, chicken, duck, goat, sheep and pork DNA in food products. Food Addit. Contam. Part A.

[18] Kim M.-J., Kim H.Y. (2018). Development of a fast duplex real-time PCR assay for simultaneous detection of chicken and pigeon in raw and heat-treated meats. Food Control.

[19] Amaral J.S., Santos G., Oliveira M.B.P.P., Mafra I. (2017). Quantitative detection of pork meat by EvaGreen real-time PCR to assess the authenticity of processed meat products. Food Control.

[20] Soares S., Amaral J.S., Oliveira M.B.P.P., Mafra I. (2013). A SYBR Green real-time PCR assay to detect and quantify pork meat in processed poultry meat products. Meat Sci..

[21] Köppel R., van Velsen F., Ganeshan A., Pietsch K., Weber S., Graf C., Murmann P., Hochegger R., Licina A. (2020). Multiplex real-time PCR for the detection and quantification of DNA from chamois, roe, deer, pork and beef. Eur. Food Res. Technol..

[22] Fajardo V., González I., Martín I., Rojas M., Hernández P.E., García T., Martín R. (2008). Real-time PCR for detection and quantification of red deer (*Cervus elaphus*), fallow deer (*Dama dama*), and roe deer (*Capreolus capreolus*) in meat mixtures. Meat Sci..

[23] Druml B., Hochegger R., Cichna-Markl M. (2015). Duplex real-time PCR assay for the simultaneous determination of the roe deer (*Capreolus capreolus*) and deer (sum of fallow deer, red deer and sika deer) content in game meat products. Food Control.

[24] Druml B., Kaltenbrunner M., Hochegger R., Cichna-Markl M. (2016). A novel reference real-time PCR assay for the relative quantification of (game) meat species in raw and heat-processed food. Food Control.

[25] Kaltenbrunner M., Hochegger R., Cichna-Markl M. (2018). Tetraplex real-time PCR assay for the simultaneous identification and quantification of roe deer, red deer, fallow deer and sika deer for deer meat authentication. Food Chem..

[26] Druml B., Uhlig S., Simon K., Frost K., Hettwer K., Cichna-Markl M., Hochegger R. (2021). Real-time PCR Assay for the detection and quantification of roe deer to detect food adulteration—Interlaboratory validation involving laboratories in Austria, Germany, and Switzerland. Foods.

[27] Adenuga B.M., Biltes R., Villa C., Costa J., Spychaj A., Montowska M., Mafra I. (2025). Unravelling red deer (*Cervus elaphus*) meat adulteration in gourmet foods by quantitative real-time PCR. Food Control.

[28] Kaltenbrunner M., Hochegger R., Cichna-Markl M. (2018). Red Deer (*Cervus elaphus*)-specific real-time PCR assay for the detection of food adulteration. Food Control.

[29] Kaltenbrunner M., Hochegger R., Cichna-Markl M. (2018). Development and validation of a fallow deer (*Dama dama*)-specific TaqMan real-time PCR assay for the detection of food adulteration. Food Chem..

[30] Liu G., Luo J., Xu W., Li C., Guo L. (2023). A novel triplex real-time PCR method for the simultaneous authentication of meats and antlers from sika deer (*Cervus nippon*) and red deer (*Cervus elaphus*). J. Food Compos. Anal..

[31] Druml B., Mayer W., Cichna-Markl M., Hochegger R. (2015). Development and validation of a TaqMan real-time PCR assay for the identification and quantification of roe deer (*Capreolus capreolus*) in food to detect food adulteration. Food Chem..

[32] Costa J., Amaral J.S., Grazina L., Oliveira M.B.P.P., Mafra I. (2017). Matrix-normalised real-time PCR approach to quantify soybean as a potential food allergen as affected by thermal processing. Food Chem..

[33] Bustin S.A., Benes V., Garson J.A., Hellemans J., Huggett J., Kubista M., Mueller R., Nolan T., Pfaffl M.W., Shipley G.L. (2009). The MIQE Guidelines: Minimum information for publication of quantitative real-time PCR experiments. Clin. Chem..

[34] ENGL (European Network of GMO Laboratories) (2015). Definition of Minimum Performance Requirements for Analytical Methods of GMO Testing. European Network of GMO Laboratories, Join Research Centre, EURL. https://gmo-crl.jrc.ec.europa.eu/doc/MPR%20Report%20Application%2020_10_2015.pdf.

[35] Ahmad Nizar N.N., Hossain M., Sultana S., Ahamad M.N., Johan M.R., Ali M.E. (2019). Quantitative duplex real-time polymerase chain reaction assay with TaqMan probe detects and quantifies *Crocodylus porosus* in food chain and traditional medicines. Food Addit. Contam. Part A.

[36] Chavan S.D., Desai D.M. (2022). Analytical method validation: A brief review. World J. Adv. Res. Rev..

