# Supplementary material for: A Novel Normalized Quantitative Real-Time PCR Approach for Ensuring Roe Deer (Capreolus capreolus) Meat Authenticity in Game Meat Foods
Source: Foods. 2024 Nov 21;13(23):3728. doi: 10.3390/foods13233728 (PMC11640146; doi:10.3390/foods13233728)
Supplement: Supplementary file 1 [file foods-13-03728-s001.zip › foods-3310576-supplementary-v5.pdf]

Supplementary Material

A novel Normalized Quantitative Real-Time PCR Approach for Ensuring Roe Deer (*Capreolus capreolus*) Meat Authenticity in Game Meat Foods

Bukola M Adenuga [1, 2], Rita Biltes [1], Caterina Villa [1], Joana Costa [1], Anita Spychaj [2], Magdalena Montowska [2], and Isabel Mafra [1,*]

[1] REQUIMTE-LAQV, Faculdade de Farmácia, Universidade do Porto, Rua de Jorge Viterbo Ferreira, 228, 4050-313, Portugal

[2] Department of Meat Technology, Poznań University of Life Sciences, ul. Wojska Polskiego 31, 60-624 Poznań, Poland; bukola.adenuga@up.poznan.pl

* Correspondence: isabel.mafra@ff.up.pt

Table S1. Results of qualitative PCR targeting a universal eukaryotic region of the 18S rRNA gene and the agouti signaling protein (ASIP) gene of roe deer using several relevant animal and plant species for cross-reactivity testing.

**Common name**	**Scientific name**	**Qualitative PCR**	
		18SRG-F/18SRG-R	ASIP-F/ASIP-R
Roe deer	*Capreolus capreolus*	+	+
Red deer	*Cervus elaphus*	+	+
Fallow deer	*Dama dama*	+	+
Chicken	*Gallus gallus*	+	-
Beef	*Bos taurus*	+	-
Horse	*Equus ferus caballus*	+	-
Pheasant	*Phasianus colchicus*	+	-
Lamb	*Ovis aries*	+	-
Goat	*Capra hircus*	+	-
Duck	*Anas platyrhynchos*	+	-
Guinea fowl	*Numida meleagris*	+	-
Quail	*Coturnix coturnix*	+	-
Pig	*Sus scrofa domestica*	+	-
Rabbit	*Oryctolagus cuniculus*	+	-
Pigeon	*Columba livia*	+	-
Turkey	*Meleagris gallopavo*	+	-
Wild boar	*Sus scrofa*	+	-
Soybean	*Glycine max*	+	-
Pine nut	*Pinus species*	+	-
Wheat	*Triticum aestivum*	+	-
Rosemary	*Rosmarinus officinalis*	+	-
Mustard	*Brassica juncea*	+	-
Coriander	*Coriandrum sativum*	+	-
Onion	*Allium cepa*	+	-
Garlic	*Allium sativum*	+	-
Parsley	*Petroselinum crispum*	+	-
Bay leaves	*Laurus nobilis*	+	-
Sage	*Salvia officinalis*	+	-
Fennel	*Foeniculum vulgare*	+	-
Vine	*Vitis vinifera*	+	-
Olive	*Olea europaea*	+	-
Ginger	*Zingiber officinale*	+	-
Oregano	*Origanum vulgare*	+	-
White pepper	*Piper nigrum*	+	-
Sweet chilli	*Capsicum annuum*	+	-
Chilli	*Capsicum* spp	+	-
Corn	*Zea mays*	+	-

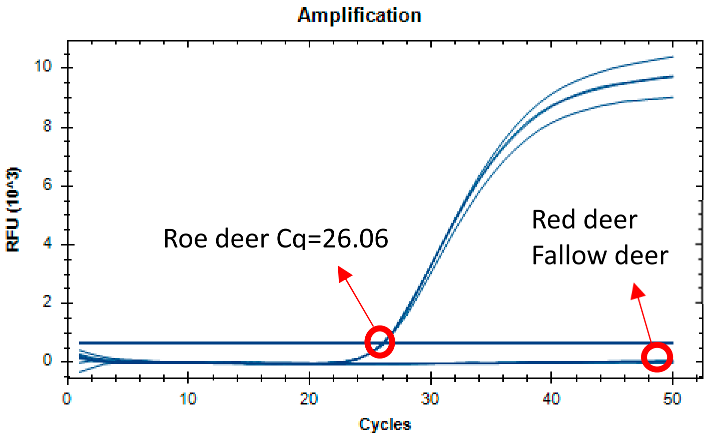

Fig. S1. Amplification curves obtained by real-time PCR with a TaqMan probe targeting the ASIP gene using DNA extracts of roe deer, red deer and fallow deer at the same concentration (50 ng/μL).

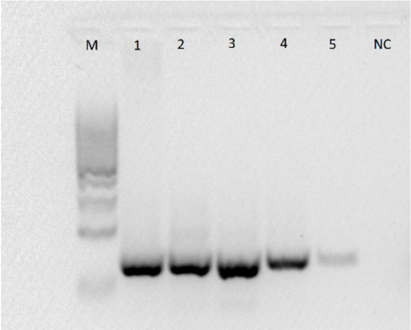

Fig. S2. Agarose gel electrophoresis of PCR products targeting agouti signalling protein (ASIP) gene of roe deer using serially diluted roe deer DNA. Legend: M, 100 bp DNA Ladder; lane 1, 40 ng; lane 2, 4 ng; lane 3, 0.4 ng; lane 4, 0.04 ng; lane 5, 0.004 ng; NC, negative control.

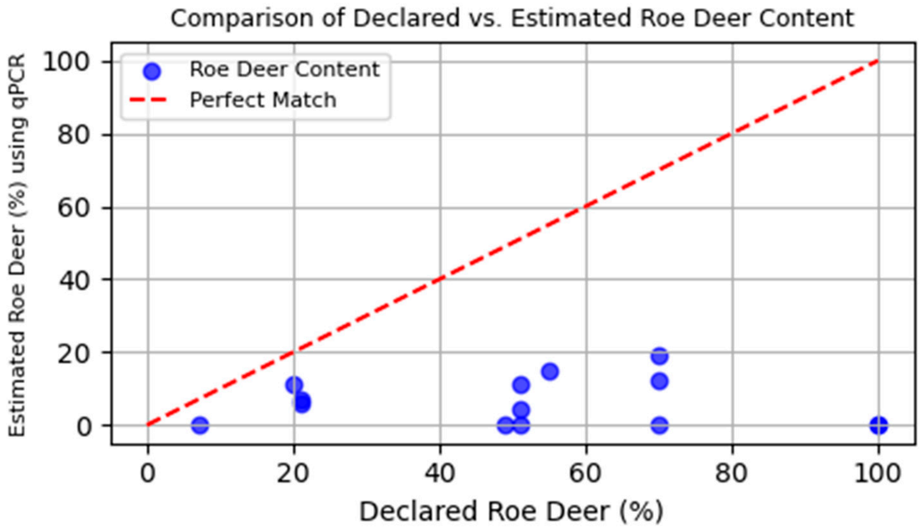

Fig. S3. Comparison of declared *versus* estimated roe deer contents by real-time PCR.
